# Integrating local knowledge and remote sensing for eco-type classification map in the Barotse Floodplain, Zambia

**DOI:** 10.1016/j.dib.2018.07.009

**Published:** 2018-07-09

**Authors:** Trinidad Del Rio, Jeroen C.J. Groot, Fabrice DeClerck, Natalia Estrada-Carmona

**Affiliations:** a University of Twente, The Netherlands; b Wageningen University & Research, The Netherlands; c Bioversity International, France

**Keywords:** Thematic map, Landsat-8 satellite data, Barotseland, Vegetation types, Geographical distribution, GIS

## Abstract

This eco-type map presents land units with distinct vegetation and exposure to floods (or droughts) in three villages in the Barotseland, Zambia. The knowledge and eco-types descriptions were collected from participatory mapping and focus group discussions with 77 participants from Mapungu, Lealui, and Nalitoya. We used two Landsat 8 Enhanced Thematic Mapper (TM) images taken in March 24th and July 14th, 2014 (path 175, row 71) to calculate water level and vegetation type which are the two main criteria used by Lozi People for differentiating eco-types. We calculated water levels by using the Water Index (WI) and vegetation type by using the Normalized Difference Vegetation Index (NDVI). We also calculated the Normalized Burn Ratio (NBR) index. We excluded burned areas in 2014 and built areas to reduce classification error. Control points include field data from 99 farmers’ fields, 91 plots of 100 m^2^ and 65 waypoints randomly selected in a 6 km radius around each village. We also used Google Earth Pro to create control points in areas flooded year-round (e.g., deep waters and large canals), patches of forest and built areas. The eco-type map has a classification accuracy of 81% and a pixel resolution of 30 m. The eco-type map provides a useful resource for agriculture and conservation planning at the landscape level in the Barotse Floodplain.

## Specifications Table

**Table:** 

Subject area	Earth Science, Environmental sciences, social sciences
More specific subject area	Remote Sensing, GIS, Landscape Ecology
Type of data	Raster (Geotiff), Vector (shapefile)
How data was acquired	Collected from the field and download from NASA and USGS website
Data format	Analyzed
Experimental factors	Image processing
Experimental features	Image classification, combined satellite data and local knowledge data in GIS using ArcGIS 10.2 and ERDAS imagine software
Data source location	Eco-type local knowledge and control points located around Mapungu, Lealui and Nalitoya villages in the Barotse Floodplain, Zambia
Data accessibility	Data is in this data article

## Value of the data

• The proposed methodology creates useful and relevant spatial information for inhabitants, decision-makers, and researchers.
• The eco-type map could facilitate guiding conservation efforts and research on habitat for aquatic and forest-dependent species.
• The eco-type map could facilitate guiding agriculture research and development efforts in the eco-types with low conservation value.

## 1 Data

The data presented herein show the eco-type classification in 2014 for the Barotse Floodplain. The eco-type was constructed by integrating Lozi People knowledge, field data and remote sensing.

## 2 Experimental design, materials and methods

### 2.1 Plot sampling and waypoints

We surveyed and geo-located ninety-one 10 × 10 m^2^ plots within a six km radius around each community between July 23rd and August 16th, 2014. We limited sampling to areas that remained unflooded or were flooded with water to a height of less than 50 cm. Recorded information included the eco-type name (based on local knowledge and names in Lozi, the local language), geographic coordinates and land cover. We collected an additional 65 waypoints which only recorded the local eco-type name and the coordinates. We used plots and waypoints for the accuracy assessment.

### 2.2 Farmers field and high-resolution imagery in Google Earth Pro

We characterized 99 farmer׳s fields across communities (4 in Lealui, 4 in Mapungu and 5 in Nalitoya). Field sizes ranged from 445 m^2^ to 2.44 ha. The centroid of each field was used as training data for the July image classification. We also used Google Earth Pro imagery to create training points on deep water, patches of forest and built areas.

### 2.3 Landsat imagery pre-processing

We analyzed two Landsat 8 Enhanced Thematic Mapper (TM) images from March 24th and July 14th, 2014 (path 175, row 71). The selected March and July images had the lowest cloud coverage and highest quality during the flooded and fieldwork period. The flooded period usually spans from February until May [1]. Fieldwork took place during mid-July and beginning of August which overlaps with the cold period (May–August) of the dry season (May– November) [1], [2]. We applied a simple dark object subtraction (DOS) correction to both images for amending atmospheric scattering and absorption and for accurately estimating surface reflectance [3] using ERDAS Imagine 13.0.2.

### 2.4 Sub-areas for land type classification

**Fig. 1:**
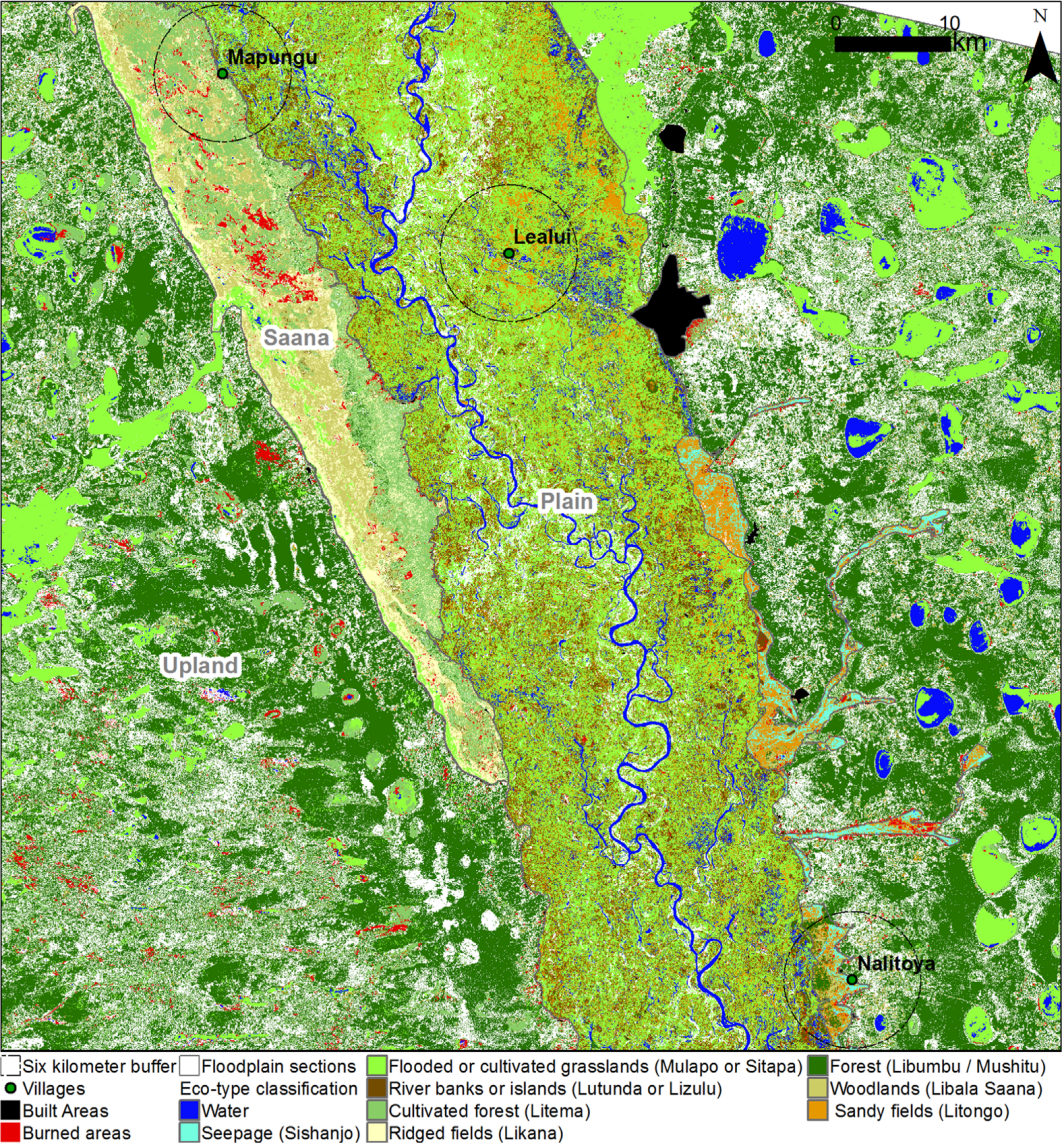
Eco-type classification in 2014 for the Barotse Floodplain. Please refer to Table 1. for eco-type description.

According to Lozi knowledge [4], eco-type characteristics are determined by their location along the floodplain either in the (1) Floodplain, (2) Saana (seepage) or (3) Upland area (Fig. 1). We used Google Earth Pro to delimit each section during the participatory activities. Subsequently, we classified dry and wet areas during the flooded period using the water index (WI) and the Landsat image in March 2014 (middle of the flooding period). Control points for the cut-off value included 15 water-related waypoints (canals, rivers or ponds), 30 plots with grasslands still flooded during the fieldwork in July–August and 200 points in areas flooded year-round (e.g., deep waters and large canals) from Google Maps Pro. We used the dry and wet areas during the flooded period as a surrogate for elevation due to the lack of high-resolution digital elevation model for the area and minimal elevational differences in the very flat floodplain.

### 2.5 Indexes

We calculated the Normalized Burn Ratio (NBR) index to identify recently burned areas. We excluded burned areas from the eco-type classification since both; the NDVI (a vegetation-based metric, see below) and the Water Index are affected by fires. Slash and burn is a common practice in the region [5]. Natural grasslands (*Mulapos*) and forest (*Mushitu*) are often converted to cropland after floods reside [4]. The NBR was calculated with the near-infrared and shortwave-infrared reflectance ratio [NBR = (NIR − SWIR 2)/(NIR + SWIR 2)]. The NBR accurately has been demonstrated to detect burned areas with Landsat 8 images [6]. We calculated NBR for the July image and visually compared cut-off values displaying SWIR 2-NIR-coastal aerosol band combination (7-5-1 RGB).

We used the Water Index (WI) and the Normalized Difference Vegetation Index (NDVI) to classify eco-types. Vegetation and flooding patterns are two main factors (together with soil fertility) used by Lozi People to differentiate eco-types. The Water Index (WI) is the addition of the near-infrared and mid-infrared bands [WI = NI + SWIR 2] which is a simple and efficient method for mapping flood extent [7]. The Reflective infrared band helps to delineate land and water boundaries whereas the mid-infrared band helps to reduce potential confusion between water (low reflectance), asphalt (intermediate reflectance) and other dry areas (high reflectance). Pixels with low WI values indicate flooded areas whereas high WI values are non-aquatic or dry areas [7].

The Normalized Difference Vegetation Index (NDVI) was calculated using the red and near-infrared reflectance ratio [NDVI = (NIR − RED)/(NIR + RED)]. Chlorophyll absorbs red whereas the mesophyll leaf structure scatters near-infrared. NDVI values close to − 1 (dark) indicate that vegetation is absent and values close to 1 (light) indicate that vegetation is actively photosynthesizing (chlorophyll abundance) [8], [9].

### 2.6 Land type classification methods and accuracy assessment

**Fig. 2:**
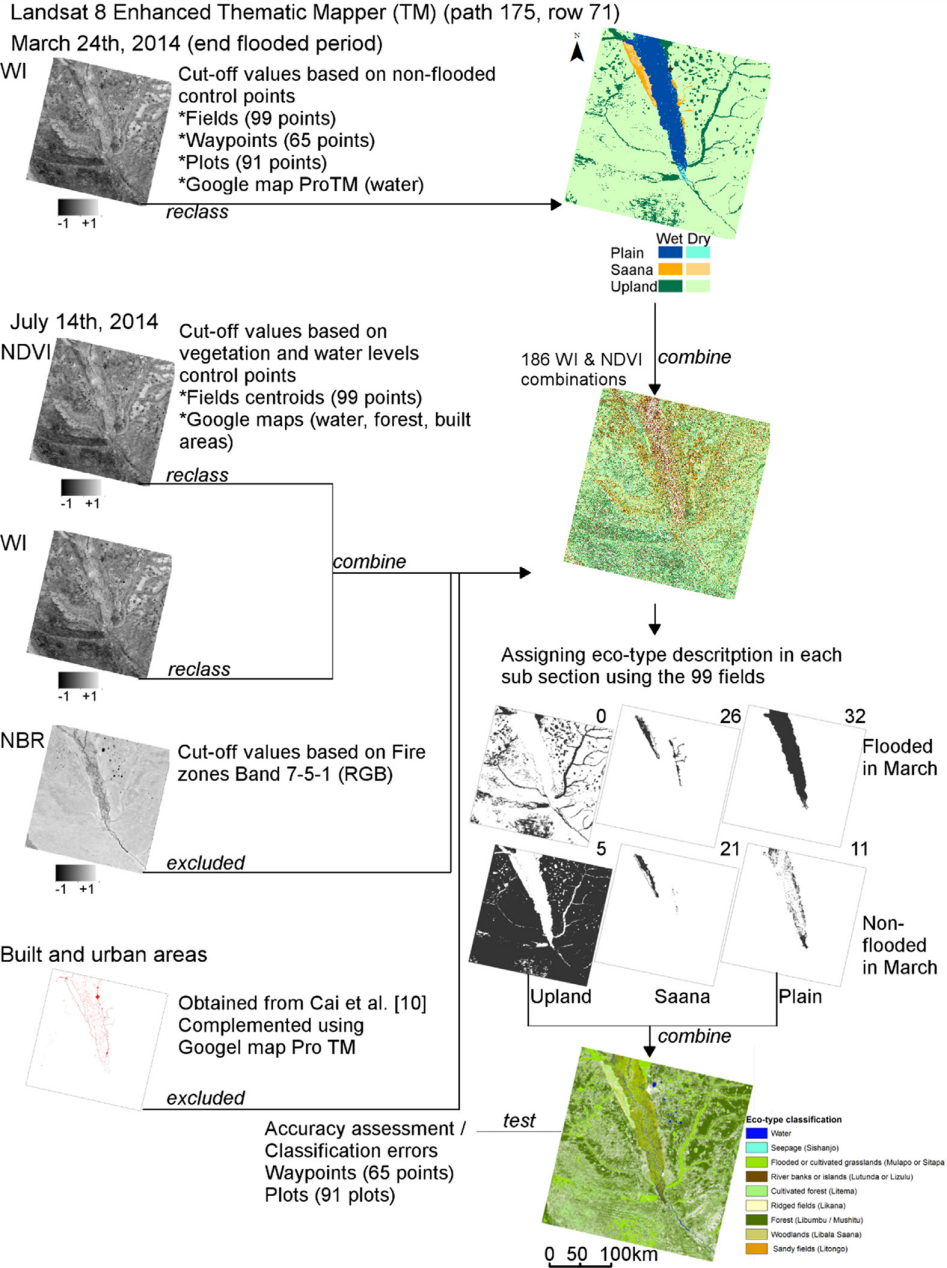
Experimental design. March image was used to classify flooded and non-flooded areas as well as the three main sections of the floodplain: Plain, Saana, Upland. The July image was used to calculate the NDVI and WI values. The resulting combination of NDVI and WI values was used to assign the eco-types in each subsection. Recently burned (NBR) and built areas were excluded from the classification.

We complemented the built areas file produced by [10]. We added other permanent villages using Google Earth Pro. These built areas and burned areas were excluded before the eco-type classification. We joined the WI and NDVI classified raster files obtaining 186 different combinations of water levels and vegetation types. We used the 99 farmer׳s field information for matching the different combinations with the eco-types descriptions and locations. The eco-type assignment was conducted independently in each section for the flooded and non-flooded areas (Fig. 2).

The 91 plots and 65 waypoints served for conducting the accuracy assessment, calculating the error matrix and kappa coefficient (K_hat_) [11], [12]. The classified map (~ 72.1% of the tile) had an overall probability of 81% for correctly classifying the nine eco-types and a 78% better agreement than a classification by chance alone (Kappa Coefficient) (Table 2). Two eco-types dominated by natural vegetation but often converted to agriculture were the most dominant along the floodplain, *Libumbu/Mushitu* and *Mulapo/Sitapa. Mulapo/Sitapa* and Water were the eco-types with the highest commission error of 25% each, indicating that the areas of these ecotypes were the most overestimated. On the contrary, *Litongo* area was the most underestimated as indicated by the highest omission error (36%). The excluded burned and built area represented 1.84% (689.7 km^2^) and 0.16% (61.6 km^2^) of the tile respectively, whereas 25.85% (9703.6 km^2^) of the tile area remained as unclassified since these areas represents other eco-types than those described by local communities and verified during the field work (Tables 1 and 2).

**Table 1:** Eco-type name (in the Lozi language) and description obtained from participatory mapping and focus group discussions with 77 participants from Mapungu, Lealui, and Nalitoya. Area represents the estimated extent of the eco-type in the map.

Lozi name	Approximate english translation	Description (Floodplain section/Flood exposure)	Area (km^2^)	% area
*Libumbu/Mushitu*	Lowland forest/Upland Forest	Lowland forest often located on Islands [*Mazulu*]. Very little remains. Only mentioned in Mapungu (Plain/Moderate). Upland forest with different human intervention levels and degradation levels (Upland/Null)	16,370.3	60.2
*Mulapo/Sitapa*	Flooded grassland/Cultivated grasslands	Mulapo: Concave area often with aquatic grass. First land to become flooded and the last to dry out (Plain, Saana/High); Sitapa: Refers to a cultivated Mulapo, planted in July–Aug after flood waters recede. Cultivated crops must have a very short growing period (< 5 months) or resistance to flooded conditions (Found in the Plain, Saana, Upland/High)	5437.5	20.0
*Litema*	Cultivated forest	Cultivated upland forest, *Mushitu,* with low vegetation density. Planted in Aug/Sep (Upland/Null)	2908.4	10.7
*Wet/dry Litongo*	Wet or dry sandy fields	Wet sandy fields flood under high floods, and crop yield depended on rain, residual moisture and incorporated organic matter. Planted in May/Jun or Aug–Oct/Nov (Plain, Saana/Low). Dry sandy fields similar to wet Litongo except it does not get flooded. Planted in Aug or Oct/Nov (Plain, Saana/Null)	745.9	2.7
*Lutunda/Lizulu*	Riverbanks/Islands	Past or recent River banks deposits with an elongated shape. Riverbanks deposits are areas slightly elevated but can get flooded depending on its size and location. Planted in Aug–Oct in Mapungu or May–Dec in Lealui (Plain, Saana/Moderate). Islands often human-made and circular shaped. It can get flooded depending on its size. Planted in Nov/Dec when the rainy season starts or earlier if closer to the water (Plain, Saana/High – Moderate)	663.52	2.4
Water	Water (River/Canals/Permanent and ephemeral ponds)	The Zambezi river and major branches (Plain). The canals form a complex network across the Floodplain. Often poorly maintained. Used for transportation, irrigation and clearing land for agriculture. Have high cultural values (Plain, Saana)	576.8	2.1
*Libala Saana*	Woodlands	Woodland with sparse and short trees which are cut (some) to plant crops (mostly cassava). It floods under high floods. Planted in Nov/Dec with the onset of the raining season or earlier if closer to water (e.g., Aug/Sep) (Saana/Low)	166.8	0.6
*Likaña*	Ridged fields	Ridged area to drain water during the raining season. Planted in Apr/May at the end of the rainy season. Only mentioned to be planted in Mapungu (Saana/Low)	151.0	0.6
*Sishanjo*	Seepage	At the Floodplain׳s edge (*Mukulo*). This seepage receives underground water from upland ponds, adjacent canals, and the River. For instance, cropping activities depend on canal maintenance. Planted in Aug/Oct or Apr. Only mentioned to be planted in Nalitoya (Saana/High – Moderate).	56.0	0.2
**Total area**			**27,076.3**	**100**

**Table 2:** Barotse floodplain eco-type map accuracy assessment, error matrix.

Reference or ground truth classes												
Land Types Classification	*Libumbu/Mushitu*	*Mulapo/Sitapa*	*Litema*	*Wet/dry Litongo*	*Lutunda/Lizulu*	Water	*Libala Saana*	*Likaña*	*Sishanjo*	Total Pixels	User׳s Accuracy	Commission error
*Libumbu/Mushitu*	7									7	1.00	0.00
*Mulapo/Sitapa*		38		7	6					51	0.75	0.25
*Litema*		1	8				1			10	0.80	0.20
*Wet/dry Litongo*				21	2	1				24	0.88	0.13
*Lutunda/Lizulu*		4		4	32					40	0.80	0.20
Water		1				3				4	0.75	0.25
*Libala Saana*				1			7			8	0.88	0.13
*Likaña*								2		2	1.00	0.00
*Sishanjo*							1		9	10	0.90	0.10
Total Pixels	7	44	8	33	40	4	9	2	9	156		
Producer׳s Accuracy	1.00	0.86	1.00	0.64	0.80	0.75	0.78	1.00	1.00		**0.81**	
Omission error	0.00	0.14	0	0.36	0.20	0.25	0.22	0.00	0.00			
